# Multiscale Entropy Analysis: Application to Cardio-Respiratory Coupling

**DOI:** 10.3390/e22091042

**Published:** 2020-09-18

**Authors:** Mirjana M. Platiša, Nikola N. Radovanović, Aleksandar Kalauzi, Goran Milašinović, Siniša U. Pavlović

**Affiliations:** 1 Institute of Biophysics, Faculty of Medicine, University of Belgrade, KCS, PO Box 22, 11129 Belgrade, Serbia; 2 Pacemaker Center, Clinical Center of Serbia, 11000 Belgrade, Serbia; nikolar86@gmail.com (N.N.R.); goran@milasinovic.com (G.M.); pavlosini@yahoo.com (S.U.P.); 3 Department for Life Sciences, Institute for Multidisciplinary Research, University of Belgrade, 11000 Belgrade, Serbia; kalauzi.alex@gmail.com; 4 Faculty of Medicine, University of Belgrade, 11000 Belgrade, Serbia

**Keywords:** cross multiscale entropy analysis, sample entropy, cardiopulmonary coupling, heart rhythm, respiratory rhythm, heart failure, atrial fibrillation, sinus rhythm, sinus rhythm with ventricular extrasystoles, autonomic nervous system

## Abstract

It is known that in pathological conditions, physiological systems develop changes in the multiscale properties of physiological signals. However, in real life, little is known about how changes in the function of one of the two coupled physiological systems induce changes in function of the other one, especially on their multiscale behavior. Hence, in this work we aimed to examine the complexity of cardio-respiratory coupled systems control using multiscale entropy (MSE) analysis of cardiac intervals MSE (RR), respiratory time series MSE (Resp), and synchrony of these rhythms by cross multiscale entropy (CMSE) analysis, in the heart failure (HF) patients and healthy subjects. We analyzed 20 min of synchronously recorded RR intervals and respiratory signal during relaxation in the supine position in 42 heart failure patients and 14 control healthy subjects. Heart failure group was divided into three subgroups, according to the RR interval time series characteristics (atrial fibrillation (HFAF), sinus rhythm (HFSin), and sinus rhythm with ventricular extrasystoles (HFVES)). Compared with healthy control subjects, alterations in respiratory signal properties were observed in patients from the HFSin and HFVES groups. Further, mean MSE curves of RR intervals and respiratory signal were not statistically different only in the HFSin group (*p* = 0.43). The level of synchrony between these time series was significantly higher in HFSin and HFVES patients than in control subjects and HFAF patients (*p* < 0.01). In conclusion, depending on the specific pathologies, primary alterations in the regularity of cardiac rhythm resulted in changes in the regularity of the respiratory rhythm, as well as in the level of their asynchrony.

## 1. Introduction

Over the past few years, the quantification of the various types of interactions of complex physiological systems and the properties of their own regulatory mechanisms, in physiological and pathological states, has become a challenge for many physicists and mathematicians [1,2]. The most explored interactions of the cardiovascular and respiratory system, as an example of the interaction of two complex physiological systems, have recently become of great interest [2,3,4,5,6,7].

The approximate entropy and its refined version known as sample entropy, have been used in analysis of biosignals to estimate the degree of randomness or regularity in physiological processes [7,8,9,10,11,12,13], including patterns of hormonal secretion, EEG, heart rate, and respiratory dynamics. These entropy measures quantified the regularity of the data reflecting the correlation properties of the time series. Furthermore, cross-approximate entropy, and its improved version cross-sample entropy, are measures of asynchrony between two paired time series, and they are usually applied to compare sequences of distinct intertwined variables in a network, estimating uncoupling, or changes in feedback regulatory mechanisms [6,7,8,13,14].

It is known that biological systems are integrated systems that function at multiple time scales. Usually, biosignals are multiscaled, and their properties depend on the scale at which the signals are analyzed. Taking this into account, Costa and coauthors [15,16] developed a multiscale entropy (MSE) analysis—an estimation of entropies from the whole set of coarse-grained time series with the goal to quantify data complexity. Their findings revealed that entropy measures depend on time scale determined features of the regulatory mechanisms of the analyzed system. Since its introduction, numerous modifications and refinements of the original MSE algorithm have been proposed [17,18,19,20,21]. In general, they are based on alternative coarse-graining procedures and reduction of the decrease of variation with increase of scale factor. This method has also been extended to multiscale permutation entropy analysis [22], and multivariate multiscale entropy analysis [23,24]. In previous studies, the difference between MSE curves has not been quantified.

Various properties of physiological signals carry information about the state of systems as well as the state of their regulatory mechanisms. The aim of this study was to assess the regularity of cardiac and respiratory control via MSE analysis of heartbeat intervals and respiratory signal, and the synchrony between these time series by cross MSE analysis in heart failure patients. Understanding the behavior of coupled physiological systems in pathological conditions through the analysis of coupled physiological signals can reveal the existence and level of possible developed compensatory mechanisms that could be complementary to common clinical parameters.

## 2. Materials and Methods

### 2.1. Participants and Data Collection

**Figure 1:**
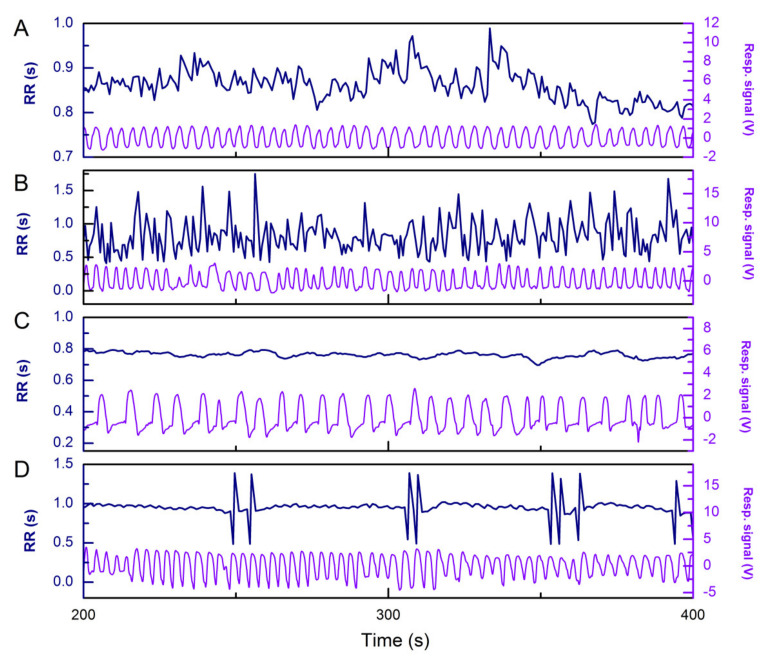
Examples of RR interval time series and respiratory signal in a one healthy subject (**A**), and heart failure patients in the case of atrial fibrillation (**B**), sinus rhythm (**C**) and sinus rhythm with ventricular extrasystoles (**D**).

Experiments were done early in the morning in a quiet surrounding at the Pacemaker Center, Clinical Center of Serbia. The study was conducted in accordance with the Declaration of Helsinki, and the protocol was approved by the Ethics Committee of the Faculty of Medicine the University of Belgrade (Approve Date: 17th March 2017, Ref. Numb. 29/III−4). All subjects gave their informed consent for inclusion before they participated in the study. We included symptomatic heart failure (HF) patients with reduced left ventricular ejection fraction (LVEF < 35%). This population was divided into three groups by the properties of their RR interval time series (Figure 1): with atrial fibrillation (HFAF), with sinus rhythm (HFSin), and sinus rhythm with ventricular extrasystoles (HFVES) (approximately 60 VES per subject during a 20 min period). The control group consisted of healthy subjects who had no previous history of any disease. Characteristics of included patients and control subjects are presented in Table 1.

All subjects underwent 20 min of simultaneous ECG and respiration measurement in supine position at spontaneous breathing frequency, after 15 min of relaxation. Both signals, ECG and respiratory, were acquired with 1000 Hz sampling frequency by Biopac MP100 system with AcqKnowledge 3.9.1. software (BIOPAC System, Inc., Santa Barbara, CA, USA). Respiratory signal was recorded via transducer attached to the belt, which is used to measure abdominal expansion and contraction. OriginPro 8.6 (OriginLab Corporation, Northampton, MA, USA) tool was used to extract interbeat (RR) intervals from ECG and to form the time series of RRs. The respiratory signal was low pass filtered with the cutoff frequency of 1 Hz. Since the respiratory signal was uniformly sampled with sampling frequency of 1 kHz, while samples of RR were unequally positioned, equal equidistant resampling of Resp and RR signal was done with our original MATLAB R2010a (Version 7.10.0.499) program using the mean value of RR for each subject data [13]. The mean RR interval was calculated from a 20 min RR interval time series for each person. This value was used to resample both RR interval series and respiratory signal for this subject, in order to obtain two comparable series with an equal number of equidistant samples. The resampling procedure was performed by linear interpolation between two corresponding adjacent existing samples.

### 2.2. Sample Entropy, Multiscale Entropy, and Cross Multiscale Entropy

Sample entropy (SampEn) is a measure of time series irregularity (unpredictability) over single scale. It was developed by Richman and Moorman [9] as a refinement of the approximate entropy introduced by Pincus (1991) [8]. For some time series, SampEn is defined as the negative natural logarithm of the conditional probability that two sequences, similar for *m* points, remain similar within tolerance *r* at the next point (i.e., for *m* + 1 points), where self-matches are not included:

(1) $$\mathrm{SampEn}(N,m,r)=-\ln\frac{A^{m}(r)}{B^{m}(r)}$$

where *B^m^(r)* is the probability that two sequences will match for *m* points, whereas *A^m^(r)* is probability that two sequences will match for *m* + 1 points, *N* is the number of equidistant data points. The time series with a small number of similarities is characterized by large values of sample entropy, which indicates its higher unpredictability (irregularity).

Multiscale entropy (MSE) analysis is a method in which by calculation of sample entropy of coarse-grained time series overall complexity of the original time series is quantified. This method has two steps. Firstly, coarse-grained time series of RR interval and respiratory signals were obtained by averaging the data points within non-overlapping windows of increasing length *τ*, the length of coarse grained series becoming *N*/*τ*, as in the original approach of MSE [15]. Secondly, we calculated sample entropy for each set of cardiac and respiratory coarse-grained time series. The Matlab code for calculation of sample entropy used in this work was taken from PhysioNet [25]. In congruence with previous study [16], *m* = 2 and the tolerance level of *r* = 15% × SD (standard deviation) of the normalized time series (RR and Resp) were used as input parameters. In the original MSE approach proposed by Costa et al., (2002) [15] value of *r* was constant for all scale factors. Calculations were performed on the whole series from 20 min recording (approximately 1200 data points). The scale factor *τ* determines the number of data points averaged to obtain each element of the coarse-grained time series. In our work, the scale factor *τ* was in the range *τ* = (1,…, 20), and *N*/*τ* was approximately between 600 data points for *τ* = 2 and 60 data points for *τ* = 20. Furthermore, we averaged data for each scale factor *τ* and calculated their standard errors in the group. As the final result, we presented the curve of connected averaged values. Further, we statistically tested the difference between these curves in a group or between groups.

It is known that the properties of time series depend on the degree of correlation between data points. White noise is an example of random walk fluctuations with uncorrelated data (rough “landscape”). When data are correlated, precisely when long-range correlations are present, this process corresponds to 1/*f* noise. Processes where strong correlations exist but cease to be power law as in 1/*f* noise, are known as an integral of white noise, so called Brownian noise (smooth “landscape”). Costa et al., 2002 [15] tested the MSE method on simulated white and 1/*f* noise and showed that for coarse-grained 1/*f* noise time series SampEn constant over all scale factor range (approximately with SampEn values around 1.8), while, in the case of coarse-grained white noise, the entropy values monotonically decreased.

The cross-approximate entropy as a measure of asynchrony between two time series was also introduced by Pincus (1991) [8]. We used cross-sample entropy (cross-SampEn) as a measure of the asynchrony between RR interval and respiratory time series [8,9]. This measure is a non-linear measure of time series irregularity derived from the probability of finding a similarity between two signals.

Generally, for paired, normalized and equally sampled time series of length *N*, *u* = [*u*(1), *u*(2), …, *u*(*N*)] and *v* = [*v*(1), *u*(2), …, *v*(*N*)], we formed the vector sequences *x*(*i*) = [*u*(*i*), *u*(*i* + 1),…, *u*(*i* + *m* − 1)], 1 ≤ *i* ≤ *N* − *m*, and *y*(*j*) = [*v*(*j*), *v*(*j* + 1),…, *v*(*j* + *m* − 1)], 1 ≤ *j* ≤ *N* − *m*. Input parameters were defined as for MSE: *m* as template length and *r* as matching tolerance. Then, we defined the maximum distance between *x*(*i*) and *y*(*j*), d[*x*(*i*), *y*(*j*)], as the maximum absolute difference in their scalar components:

(2) $$d\left[x_{m}(i),y_{m}(j)\right]=\max\left\{\left|u(i+k)-v(j+k)\right|\right\},k=0,1,\ldots ,m-1$$

For each *i* ≤ *N* − *m*,

$$B_{i}^{m}(r)(v\left\|u\right.)\;=(\mathrm{number}\;\mathrm{of}\;1\;\leq \;j\leq \;N\;-\;m$$

such that

$$d\left[x_{m}(i),y_{m}(j)\right]\leq r)/(N-m)$$

$B_{i}^{m}(r)(v\left\|u)\right.$ is the probability that any *y_m_*(*j*) is within *r* of *x_m_*(*i*), and its average value is defined as:

(3) $$B^{m}(r)(v\left\|u\right.)=\frac{\sum_{i=1}^{N-m}B_{i}^{m}(r)(v\left\|u\right.)}{N-m}$$

Similarly,

$$A_{i}^{m}(r)(v\left\|u)\right.=(\mathrm{number}\;\mathrm{of}\;1\;\leq \;j\leq \;N\;-\;m)$$

such that

$$d\left[x_{m+1}(i),y_{m+1}(j)\right]\leq r)/(N-m)$$

and average value of $A_{i}^{m}(r)(v\left\|u)\right.$ is

(4) $$A^{m}(r)(v\left\|u\right.)=\frac{\sum_{i=1}^{N-m}A_{i}^{m}(r)(v\left\|u\right.)}{N-m}$$

Therefore, $B^{m}(r)(v\left\|u)\right.$ is the probability that the two templates match for *m* points, and $A^{m}(r)(v\left\|u)\right.$ is the probability that the two templates match for *m*+1 points.

Finally, cross-SampEn is defined as:

(5) $$\mathrm{cross}-\mathrm{SampEn}=-\ln\left\{\frac{A^{m}(r)(v\left\|u)\right.}{B^{m}(r)(v\left\|u)\right.}\right\}$$

The coupling of two signals resulting in small values of cross-sample entropy indicates high synchrony of signals i.e., high association between analyzed systems. Calculations were done with the same input parameters, *m* = 2 and the tolerance level of *r* = 15% × SD (standard deviation) of the normalized coarse-grained time series.

In this work, we used our designed Matlab script for calculation of cross multiscale entropy (CMSE (RR−Resp)) as cross sample entropy for each pair of normalized coarse-grained heart intervals (RR) and respiratory (Resp) time series.

### 2.3. Statistics

By Shapiro–Wilk test we found that all the data were normally distributed, every measure in each of four groups. A mixed design repeated measures ANOVA with one within subject factor (scale) and one between subject factor (group) was performed to find the interaction between scale and group, and main effects of group with the least significant difference (LSD) post-hoc comparisons i.e., to find difference between MSE curves of one measure between the groups (for MSE (RR), MSE (Resp) and CMSE (RR−Resp)). Two way repeated measures ANOVA with two within subject factors (measure and scale) in each analyzed group was performed, to find difference between mean MSE curves for two measures (MSE (RR) and MSE (Resp)). In both types of ANOVAs for repeated measures, a significant main effect of scale factor was revealed. A point by point comparison for each scale factor was performed by paired *t*-test (Table 2). Detailed statistical analyses were performed by one way ANOVA with multiple post-hoc (LSD test) comparisons, on mean MSE (for both time series), as well as on mean CMSE (RR−Resp), in the ranges of pooled scale factors; short scales (1–4), middle scales (5–12) and large scales (13–20). The results are given as mean ± standard error of the mean. Significance level *p* < 0.05 was used as significant. Statistical analyses were performed using the software package SPSS Statistics for Windows version 17.0, (SPSS Inc, Chicago, IL, USA).

## 3. Results

**Figure 2:**
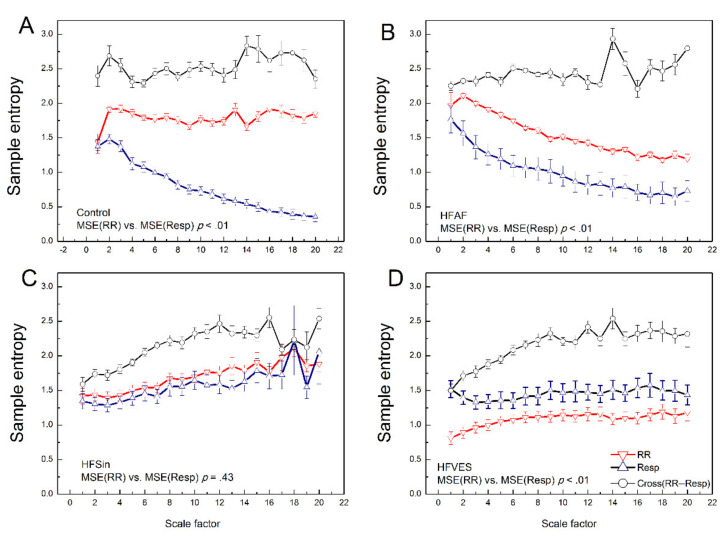
Multiscale entropy curves of RR time series, MSE (RR)—red down triangles, multiscale entropy of respiratory time series, MSE (Resp)—blue up triangles and cross multiscale sample entropy between RR and respiratory time series, CMSE—black open circles: in control healthy subjects (**A**), heart failure patients with atrial fibrillation, HFAF (**B**), heart failure patients with sinus rhythm, HFSin (**C**) and heart failure patients with ventricular extrasystoles, HFVES (**D**). Mean value ± standard error. Significance of comparison between MSE (RR) and MSE (Resp) profiles are given for each group of subjects. Detailed, point by point, comparisons for each scale factor are given in Table 2.

Figure 2 shows averaged MSE profiles for RR intervals, respiratory signal and cross RR−Resp signals obtained from connected mean sample entropy values for each coarse-grained time series at scale factor *τ* = (1,…,20).

Multiscale entropy analysis of RR interval time series, MSE (RR), revealed expected differences in MSE profiles between analyzed groups (*F* = 29.56, *p* < 0.01). Moreover, for MSE (RR), we found a significant scale × group interaction (*F* = 11.84, *p* < 0.01). Alterations of RR series regularity over different scales in heart failure patients depended on their sub-pathology.

Mean MSE (RR) curve of cardiac rhythm in control subjects and heart failure patients with sinus rhythm, appeared as MSE profile of 1/*f* correlated noise, and they were not statistically different (Figure 2A,C and Table 3). The MSE (RR) mean curve in patients with sinus rhythm and ventricular extrasystoles had the feature of strongly correlated time series with lower values of entropy (approaching Brownian noise) (Figure 2). Furthermore, profiles of MSE (RR) for heart failure patients with sinus rhythm and the HFAF group showed a tendency for statistically significant difference (Table 3).

On the other hand, multiscale entropy analysis of respiratory signal MSE (Resp) revealed unexpected differences between the analyzed groups (*F* = 11.93, *p* < 0.01) and the scale × group interaction (*F* = 7.93, *p* < 0.01). We found that there was no significant difference between MSE (Resp) curves for control subjects and HFAF patients. Their MSE profiles look like previously reported MSE profiles for simulated white noise (Figure 2, Table 3, [15]). This feature was in marked contrast to those for the MSE (Resp) curve profiles in the HFSin group and HFVES patients, which showed MSE (Resp) profiles typical for 1/*f* noise (Figure 2, Table 3).

Furthermore, by comparing MSE (RR) and MSE (Resp) mean curve profiles in the analyzed groups, we obtained the following results. Only in the control group the interaction between scale factor and measures was significant (*F* = 34.14, *p* < 0.01) (Figure 2A) i.e., cardiac 1/*f* noise was statistically different from respiratory white noise. In the heart failure groups with arrhythmias, HFAF and HFVES, the obtained profiles showed a significant difference between MSE (RR) and MSE (Resp) profiles (Figure 2). Irregularity of the cardiac rhythm in AF patients was statistically higher than irregularity of respiratory rhythm (*p* < 0.01), while respiratory rhythm had statistically greater complexity over different scales than heart rhythm in HFVES patients (*p* < 0.01). Only in the HFSin group there was no significant difference between regularity of heart and respiratory rhythm over scales of different length, where both signals had the properties of correlated 1/*f* noise (Figure 2, *p* = 0.43).

The cross multiscale entropy method was applied to examine the asynchrony between heart and respiratory rhythm by calculating the cross-sample entropy over different scales (Figure 2). Statistical analysis of CMSE (RR−Resp) mean profiles showed a significant scale × group interaction (*F* = 2.21, *p* = 0.01) and a significant main effect of the group (*F* = 17.70, *p* < 0.01). Multiple comparisons between analyzed groups showed that the asynchrony between the analyzed signals decreased from control subjects via HFAF to HFSin and HFVES patients. Post hoc multiple comparisons of CMSE (RR−Resp) profiles revealed that all analyzed groups’ profiles were significantly different, except for the CMSE (RR−Resp) profiles between the control and HFAF group, and between HFSin and HFVES patients (Table 3).

**Figure 3:**
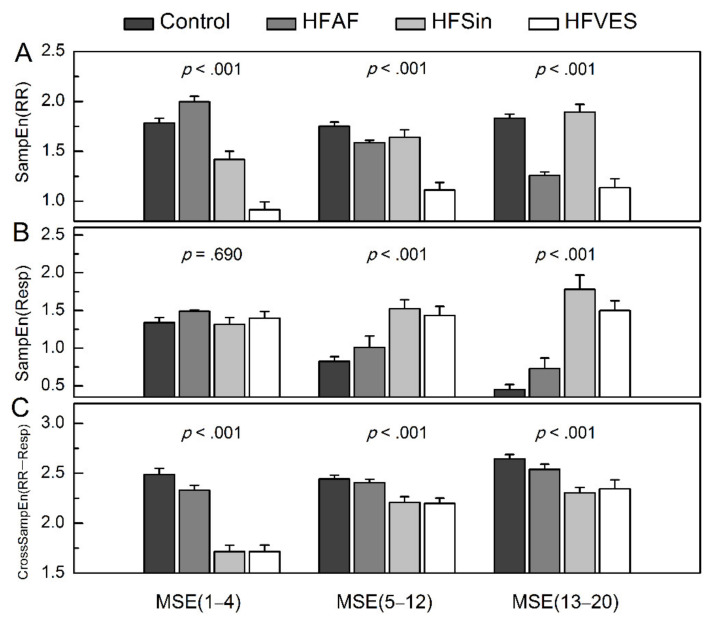
Means (plus standard errors) for sample entropy of heart rhythm, SampEn (RR), (**A**), sample entropy of respiratory rhythm, SampEn (Resp), (**B**) and cross sample entropy, CrossSampEn (RR−Resp), (**C**) over pooled scale ranges: short MSE (1–4), middle MSE (5–12), and large MSE (13–20) in healthy subjects, Control, heart failure patients with atrial fibrillation, HFAF, with sinus rhythm, HFSin, and sinus rhythm and ventricular extrasystoles, HFVES.

All previously mentioned measures were calculated in the range over short scales (1–4), middle scales (5–12) and larger scales (13–20), and then compared between the analyzed groups (Figure 3 and Table 4). The main group effect was statistically significant for all SampEn measures in all three ranges (*p* < 0.001), with the exception of SampEn (Resp) in the short range of pooled scales MSE (1–4) (*F* = 0.489, *p* = 0.690).

We found that irregularity of heart rhythm in atrial fibrillation was significantly higher than irregularity of heart rhythm in control group only over short scales MSE (1–4), and it gradually decreased over larger scales (Figure 3A). Furthermore, the regularity of HFSin patients cardiac rhythm was significantly higher than in the control group only over short pooled scales MSE (1–4), while in HFVES patients, regularity of heart rhythm was the highest, compared to other groups (Figure 3A, Table 4).

Findings from multiscale entropy analysis of respiratory rhythm showed that the main group effect was significant in the ranges of middle MSE (5–12) and larger MSE (13–20) scales (*F* = 8.370, *p* < 0.001 and *F* = 20,652, *p* < 0.001, respectively). This effect is the result of following: there is no significant difference between regularity of respiratory rhythm in control subjects and patients with HFAF and between regularity of respiratory rhythm in HFSin and HFVES patients, on both scale ranges; respiratory rhythm in HFSin and HFVES patients showed more irregular behavior on larger scales than respiratory rhythm in control subjects and HFAF patients.

The main group effect on multiscale cross sample entropy values was statistically significant over all three scale ranges: CMSE (1–4), CMSE (5–12), CMSE (13–20), (*F* = 43.018, *p* < 0.001; *F* = 6.924, *p* < 0.001; *F* = 9.506, *p* < 0.001, respectively). Asynchrony between cardiac and respiratory rhythm was higher in the control and the HFAF group than in HFSin and HFVES patients (Figure 3, Table 2). Synchrony in the last two groups was greater in the short scale ranges CMSE (1–4), than on larger scales (Table 4).

## 4. Discussion

In this work, we used a multiscale entropy analysis of cardiac and respiratory rhythm in several groups of heart failure patients. We quantify the differences between their MSE mean curve profiles and mean MSE values over three pooled scale ranges. In addition to different complexities of these rhythms between, as well as in the analyzed groups, and the findings of cross MSE profiles, we noticed that synchrony of the cardio-respiratory coupling was also scale dependent and determined by specific cardiac pathology.

In our previous work we showed that heart rhythm in heart failure patients with sinus rhythm can be grouped in four clusters, depending on the behavior over short and long scale ranges [26]. Here, we analyzed general types of cardiac rhythm alterations, and extended analysis on their relationship with respiratory rhythm. We found that there is a significant difference between mean MSE (RR) profiles of the HFAF and the control group, but cardiac rhythm in the HFAF group had similar irregularity as in healthy control subjects in the range of middle scales MSE (5–12). Moreover, it is interesting that in the HFAF group mean MSE (RR) in the three ranges of pooled scales gradually decreased, while mean MSE (RR) in control subjects was almost constant over all three scale ranges. In general, these results are in line with the findings of Costa et al. [15]. They found that MSE pattern is flat with scale factor *τ* for the 1/*f* noise and it progressively decreased with the scale factor *τ* in the case of the white noise. As is known, the absence of functionality of sinoatrial node in AF results in irregular cardiac rhythm with properties of uncorrelated white noise, while regular rhythm in healthy subjects behaved as correlated 1/*f* noise [16]. Furthermore, HFVES patients had mean MSE (RR) profile significantly lower than MSE (RR) profile in control subjects. It is characterized by higher regularity, and with detailed analysis, we showed that HFVES patients had the lowest mean sample entropies of cardiac rhythm over all three scale ranges. We supposed that the highest regularity of heart rhythm in HFVES patients resulted from superposition of altered cardiac autonomic regulation (parasympathetic withdrawal followed by increased sympathetic activity) and involvement of the additional regularity mechanism, which originated from appearances of ventricular arrhythmias. Indeed, one of the pathophysiological characteristics of heart failure is reduced heart rate variability, the beat to beat variation of the duration of the R-R interval [26]. Additionally, it is known that, in heart failure patients, premature ventricular ectopic beats do not provoke biphasic reaction of an initial acceleration and late deceleration of heart rate, as in healthy subjects [27,28]. This phenomenon, which is known as heart rate turbulence, is reduced in these patients as a result of impaired baroreflex response due to abnormal autonomic tone. It is not yet clear which compensatory mechanisms exist in ventricular arrhythmias, particularly in patients with heart failure, but it is assumed that they are partly responsible for preserved synchrony between respiratory and cardiac rhythm and for regularity of heart rate, and its significant impact on respiration in these patients. All this leads to highest heart rate regularity in HFVES patients. On the other hand, tendency of similar multiscale regularity of heart rate in healthy controls and HFSin patients where their MSE (RR) curves have in common similar values of entropies at longer scales (after fifth), can be easy explained by the existence of the autonomic nervous system imbalance, and thus heart rate variability reduction. Namely, they are significantly different over smaller scales where lower values of entropies in HFSin group indicate reduced variability resulting from the altered fast regulatory mechanism, compared to the control.

Considering the results of MSE analysis of respiratory signal MSE (Resp) in the analyzed groups with cardiovascular pathologies we found that mean MSE (Resp) profiles were statistically different between the HFAF group and the other two analyzed groups (HFSin and HFVES). Furthermore, by detailed analysis, we revealed that mean MSE (Resp) over pooled scales ranges are not different in control subjects and HFAF patients, and in HFSin and HFVES patients. According to the findings of Valencia et al. [20], we supposed that there are two types of respiratory pattern properties: one as uncorrelated white noise (as we found in HFAF and control group), and the other as correlated 1/*f* noise (as we found in HFSin and HFVES patients). Compared with the respiratory pattern in control subjects, this finding indicates unchanged regularity of the respiration pattern in AF, and altered respiratory control in HFVES and HFSin patients influenced by cardiac pathology. The presence of changes in the complexity of the respiratory rhythm in these patients is somewhat expected, due to breathing disorders, primarily Cheyne-Stokes respiration and obstructive sleep apnea, that are more often present in them, as well as higher influence of cardiac rhythm on respiratory signal in these patients, and of course, some compensatory mechanisms that are developed, but still insufficiently explored and understood [3,29,30].

Comparing MSE curve profiles of two different rhythms from coupled systems, we found a few real combinations of interactions between different types of noise in physiology and pathophysiology. In healthy subjects, we had interplay of cardiac 1/*f* noise and respiratory white noise. This finding implies that the combination of these types of noises represents some kind of rule for the optimal mechanism of interactions of complex physiological systems in healthy organism. In atrial fibrillation, we found a combination of two white noises, where the cardiac rhythm is statistically more irregular than respiratory. Since AF does not affect the regulation of the pattern of respiratory rhythm, we supposed that, besides altered autonomic control, neuroplasticity changes in the dynamics of the brainstem cardiorespiratory integrator were involved [6]. Additionally, high irregularity of cardiac rhythm in patients with atrial fibrillation is a consequence of the pathophysiological basis of the disease. It should be noted that in a previous study, we found that, in patients with atrial fibrillation, regularity of respiratory rhythm was not significantly different compared to healthy subjects [6]. In HFSin and HFVES groups both rhythms, cardiac and respiratory, were altered by pathology. We found that both rhythms in the HFSin group had properties of correlated noise (1/*f* noise) with similar complexity, and that there was a strong synchrony between them. Since the pathophysiology of heart failure is characterized by an imbalance of the autonomic nervous system, and cardio-respiratory synchronization is the result of central coupling between cardiovascular and respiratory neuronal activities, its existence should not be disturbed [31]. However, in HFVES patients, we had different changes in regulation of cardiac rhythm but similar changes in regularity of respiratory rhythm, compared to HFSin patients. Our results revealed a combination of cardiac and respiratory correlated noise (1/*f* noise), where the complexity of respiratory rhythm is higher than complexity of cardiac rhythm. This combination of two rhythms complexity should be particularly examined in further studies. It seems that the regulation of respiration pattern in HFVES has developed such a mechanism that is able to adapt to sudden changes in cardiac rhythm. This fact is supported by the highest synchrony between respiratory and cardiac rhythm in this group.

Some authors have shown that several types of cardio-respiratory interactions can coexist; besides respiratory sinus arrhythmia (modulation of heart rate by respiratory rhythm), synchronization is very often type of interaction [5,31,32,33,34]. In this work, we showed results of CMSE analysis, which was used to examine the asynchrony of coupling between cardiovascular and respiratory system, independently of influence direction. We analyzed the asynchrony between cardiac and respiratory rhythm in cardiac pathologies compared to healthy subjects, and found that CMSE curve profiles were not statistically different between control subjects and HFAF patients, and between HFSin and HFVES patients.

The lowest influence of cardiac on respiratory rhythm i.e., the highest asynchrony was revealed in healthy subjects and HFAF patients. This finding is the result of changes in RR intervals in one respiratory cycle—from the activity of RSA mechanism. By the coarse-graining technique, we eliminated the fluctuations of RR intervals on shorter scales induced by RSA, and the asynchrony was almost constant over larger scales. Contrary, the high asynchrony found in AF patients over all scale factors is an expected result, because previous findings have shown that the RSA mechanism in AF does not exist [6,34], and there is no data in the literature on the specificity of cardio-respiratory coupling in AF.

We found that in cardiac pathologies the influence of cardiac rhythm on respiration becomes significant. Compared with healthy subjects, in the HFSin and HFVES groups the highest synchrony between RR to Resp is revealed. These results imply that the influence of cardiac rhythm on respiration increased and it is related to some kind mechanisms of synchronization.

Comparing with AF, in healthy subjects the asynchrony between cardiac and respiratory rhythm is slightly higher on shorter and longer scales. This could be explained by the assumption that both types of cardio-respiratory interactions were involved, RSA over shorter and some type of synchronization on larger scales. Although we did not find a significant difference in the influence of cardiac on respiratory rhythm between healthy subjects and HFVES patients, on the short scales, the highest synchrony was detected in the HFSin and the HFVES group. This confirms altered RSA mechanism via reduced vagal control in these patients. In both groups of HF patients, with sinus rhythm or with VES, there is higher synchrony between cardiac and respiratory rhythm than in control subjects and the HFAF group over all scale factors, indicating that synchrony is the dominant mechanism of respiratory-cardio interaction in these patients. These results suggest that the influence of the altered cardiac rhythm on respiratory rhythm, measured by synchrony, depends on the scale length.

In AF, cardio-respiratory interaction is accomplished by some unknown compensatory mechanism that is not related to RSA nor synchronization, and is based on the interaction between two white noise signals. Contrary, in healthy subjects, cardio-respiratory interaction is based on the interaction of 1/*f* noise and white noise time series resulting in similar asynchrony as in AF patients. In the HFSin and HFVES group, the interaction of cardiac 1/*f* noise and respiratory 1/*f* noise resulted in stronger synchrony between rhythms, depending on the scale range, and it is highest on the short scale factors. Compared with controls, alteration of cardiac rhythm in these patients induced structural changes in the respiratory time series, especially in HFVES patients.

## 5. Conclusions

We applied particular and cross MSE analysis to RR interval and respiratory signal time series of healthy subjects and heart failure patients with three specific types of cardiac rhythm. Our findings indicate alterations in the multiscale properties of respiratory signals and their synchrony with RR interval series caused by specific cardiac rhythms in heart failure patients. Quantification of these changes in heart failure patients may have applications in monitoring alterations in regulatory mechanisms, which are usually complementary to basic clinical parameters.

## Figures and Tables

**Table 1:** Characteristics of participants.

	Control	HFAF	HFSin	HFVES
N (Females)	14 (4)	12 (4)	14	16 (4)
Age (years)	40 ± 1	68 ± 3	59 ± 3	64 ± 2
RR (s)	0.845 ± 0.033	0.835 ± 0.064	0.911 ± 0.040	0.802 ± 0.026

**Table 2:** Statistical significance of comparisons of sample entropy values for RR interval series and for respiratory signal for each scale factor *τ*.

Scale Factor, *τ*	Group			
	Control	HFAF	HFSin	HFVES
	SampEn (RR) vs. SampEn (Resp)			
1	0.675	0.513	0.620	**0.001**
2	**0.001**	**0.011**	0.302	**0.002**
3	**0.001**	**0.004**	0.433	**0.013**
4	**0.001**	**0.002**	0.435	**0.013**
5	**0.001**	**0.002**	0.487	**0.030**
6	**0.001**	**0.002**	0.533	**0.019**
7	**0.001**	**0.003**	0.355	**0.023**
8	**0.001**	**0.013**	0.494	**0.027**
9	**0.001**	**0.022**	0.519	**0.007**
10	**0.001**	**0.006**	0.766	**0.019**
11	**0.001**	**0.003**	0.304	**0.030**
12	**0.001**	**0.001**	0.335	**0.030**
13	**0.001**	**0.012**	0.071	**0.048**
14	**0.001**	**0.002**	0.335	**0.012**
15	**0.001**	**0.007**	0.528	**0.028**
16	**0.001**	**0.002**	0.801	**0.008**
17	**0.001**	**0.003**	0.427	**0.045**
18	**0.001**	**0.017**	0.834	0.061
19	**0.001**	**0.001**	0.095	**0.040**
20	**0.001**	**0.006**	0.718	0.156

Statistically significant values are in bold.

**Table 3:** Significance of least significant difference (LSD) post-hoc multiple comparisons between the groups of multiscale entropy profiles for cardiac rhythm (MSE (RR)), respiratory rhythm (MSE (Resp)), cross multiscale entropy profiles (CMSE (RR−Resp)).

	MSE (RR)	MSE (Resp)	CMSE (RR−Resp)
Control vs. HFAF	**0.007**	0.188	0.246
Control vs. HFSin	0.282	**0.001**	**0.001**
Control vs. HFVES	**0.001**	**0.001**	**0.001**
HFAF vs. HFSin	0.082	**0.001**	**0.001**
HFAF vs. HFVES	**0.001**	**0.005**	**0.001**
HFSin vs. HFVES	**0.001**	0.387	0.593

Statistically significant values are in bold.

**Table 4:** Significance of LSD post-hoc multiple comparisons between the groups of multiscale entropy measures for cardiac rhythm, MSE (RR), respiratory rhythm, MSE (Resp), and cross-sample entropy between cardiac and respiratory rhythm (CMSE) over pooled scale ranges from multiscale sample entropy patterns.

	MSE (RR) (1–4)	MSE (RR) (5–12)	MSE (RR) (13–20)	MSE (Resp) (1–4)	MSE (Resp) (5–12)	MSE (Resp) (13–20)	CMSE (1–4)	CMSE (5–12)	CMSE (13–20)
Con. vs. HFAF	**0.040**	0.083	**<0.001**	0.339	0.295	0.167	0.086	0.621	0.188
Con. vs. HFSin	**<0.001**	0.218	0.541	0.873	**<0.001**	**<0.001**	**<0.001**	**0.001**	**<0.001**
Con. vs. HFVES	**<0.001**	**<0.001**	**<0.001**	0.701	**<0.001**	**<0.001**	**<0.001**	**0.001**	**<0.001**
HFAF vs. HFSin	**<0.001**	0.571	**<0.001**	0.268	**0.004**	**<0.001**	**<0.001**	**0.008**	**0.004**
HFAF vs. HFVES	**<0.001**	**<0.001**	0.219	0.535	**0.013**	**<0.001**	**<0.001**	**0.005**	**0.011**
HFSin vs. HFVES	**<0.001**	**<0.001**	**<0.001**	0.583	0.581	0.142	0.995	0.881	0.588

Statistically significant values are in bold.
